# Intentional Training With Speech Production Supports Children’s Learning the Meanings of Foreign Words: A Comparison of Four Learning Tasks

**DOI:** 10.3389/fpsyg.2020.01108

**Published:** 2020-05-29

**Authors:** Katja Junttila, Sari Ylinen

**Affiliations:** ^1^Cognitive Brain Research Unit, Department of Psychology and Logopedics, Faculty of Medicine, University of Helsinki, Helsinki, Finland; ^2^CICERO Learning, Faculty of Educational Sciences, University of Helsinki, Helsinki, Finland

**Keywords:** foreign-language learning, incidental learning, intentional learning, cross-situational statistical learning, speech production, production effect

## Abstract

To determine the best techniques to teach children foreign words, we compared the effectiveness of four different learning tasks on their foreign-word learning (i.e., learning word forms and word meanings). The tasks included incidental learning, intentional learning with production, intentional learning without production, and cross-situational statistical learning. We also analyzed whether children’s age and cognitive skills correlate with the learning of word forms and word meanings. Forty-four 5–8-year-old children participated in the study. The results reveal that the children were able to learn the correct word forms from all four tasks and no differences emerged between the effectiveness of the tasks on the learning of word-forms. The children also learned the word meanings with all four tasks, yet the intentional task with production was more effective than the incidental task. This suggests that the ability of children to learn foreign words benefited from them knowing that they were supposed to learn new words and producing them aloud while training. The age of the children correlated with their learning results for word forms and meanings on the intentional task without production. The older children learned more effectively than the younger children in this task. Children’s phonological processing skills were correlated with learning the word meanings from the incidental task, suggesting that children with better phonological skills were able to benefit from incidental learning more than children with poorer phonological skills. Altogether, the results suggest that children’s foreign-language learning benefits from intentional training with speech production regardless of their age or cognitive skills.

## Introduction

Learning new words is an important part of foreign-language learning and it requires learning the phonological representations (word forms) and the meanings of the words (Gupta and Tisdale, 2009). Currently, learning is increasingly aided by the use of technology both in schools and outside them (Meltzoff et al., 2009). Computer games and other digital applications offer an appealing way to learn foreign languages, especially for children who are often attracted to games. This means that digital language-learning applications have the potential to enable young children to begin learning foreign languages at early ages. These applications may use tasks that require intentional or unintentional learning, speech perception or production, and direct associative learning of word-referent pairs or more indirect association through the learning of statistical co-occurrences between words and their referents. However, it is not sufficiently understood which methods would improve children’s foreign-language learning most effectively. Incidental learning (that is, learning something unintentionally^1^) and intentional learning both have been reported to be feasible methods to learn words (Ramachandra et al., 2011; Bisson et al., 2013; Archibald and Joanisse, 2013; Alipour Madarsara et al., 2015; de Vos et al., 2018), but it is not yet known whether one of them is more effective for children. Cross-situational statistical learning, referring to “learning the meanings of words across multiple exposures, despite uncertainty as to the word’s meaning on each individual exposure” (Smith and Smith, 2012), has also been demonstrated to be as effective as intentional learning without ambiguity (Venker, 2019). Production, that is, producing the words aloud during learning, has often been found to enhance learning (e.g., Hopkins and Edwards, 1972). Yet there are few studies on the production effect in children and the results are somewhat contradictory. One study determined that children benefited from producing words aloud (Icht and Mama, 2015), whereas another discovered that production hindered learning (Zamuner et al., 2018). A better understanding of the most effective methods for learning is therefore needed, for example, to develop better computer-based training programmes for language learning.

Few studies have explored children’s intentional and incidental learning of foreign words. Alipour Madarsara et al. (2015) reported that although children learned the meanings of foreign words both intentionally and incidentally, the children in the incidental group learned more words than those in the intentional group. While the actual learning tasks were identical in the two groups, the difference was that the intentional group knew they were being tested and the incidental group did not. Thus, it is debatable whether their results actually compared intentional and incidental learning tasks. Rather, the actual learning task seemed to be intentional for both groups: they were taught vocabulary with the usual methods in their schools. Their results might reflect the fact that they knew that their learning would be tested. Ramachandra et al. (2011) have also analyzed incidental learning in children by investigating their incidental learning of pseudowords with native phonemes using a story-reading within the setting of a cartoon slideshow. The analysis of that story telling reported that children with better phonological awareness performed better in learning the meanings of new words incidentally. Similarly, Abel and Schuele (2014) demonstrated that phonological awareness supported the incidental learning of unfamiliar native-language words. In addition, working memory and phonological short-term memory have been associated with more effective explicit learning of pseudoword meanings (Archibald and Joanisse, 2013).

Although few studies have been published on children’s incidental and intentional foreign-word learning, more studies have been conducted on adults (for adults’ incidental learning in speech and non-speech sounds, see Bradlow et al., 1997; Lim and Holt, 2011; Vlahou et al., 2012; Gabay and Holt, 2015; Chandrasekaran et al., 2016). Adults have been reported to learn meanings of foreign words in an incidental learning setting in which they were unaware they were tested for learning meanings (Bisson et al., 2013). Nevertheless, learning to recognize incidentally trained spoken foreign-language words is more effective than learning to recall the word meanings (de Vos et al., 2018). In an intentional training setting for adults, their attempt to retrieve a trained foreign word from memory before hearing the correct pronunciation was reported to be more effective than their trying to imitate the pronunciation after hearing the spoken word (Kang et al., 2013).

In addition to word-learning situations in which each word is unambiguously associated with its meaning, words can also be learned in cross-situational statistical settings. Cross-situational statistical learning is based on drawing conclusions from statistical regularities. One example of statistical learning is the learning of word boundaries from continuous auditory input. Saffran et al. (1996) demonstrated that infants are able to learn to distinguish words from a continuous auditory sequence of syllables by relying on statistical co-occurrences. In addition to learning to segment words from auditory sequences, word meanings can also be learned cross-situationally. This occurs through a series of individually ambiguous word-picture association trials by associating the words and pictures that statistically co-occur most often.

Cross-situational statistical learning has been demonstrated to occur when infants (Smith and Yu, 2008) and children (Suanda et al., 2014; Venker, 2019) learn native-language pseudowords that are linked to pictures of novel objects. Researchers have suggested that important factors in children’s ability to learn native pseudowords cross-situationally is their memory and language skills (Vlach and DeBrock, 2017). A recent study by Venker (2019) reported no difference between the learning of native-like pseudoword-referent pairings for the cross-situational task and an explicit task in which each pseudoword was presented only with its correct object-referent one by one. The results confirm that older children learn pseudoword-referent pairings better during cross-situational tasks than younger children. Most studies on children’s cross-situational learning have used pseudowords with native phonemes as auditory stimuli. Nonetheless, a study by Hu (2017) demonstrated that children can also learn foreign-language words for unfamiliar objects cross-situationally. Indeed, children with better phonological skills demonstrated that they were better at learning the meanings of foreign words.

Studies on adults have established that they learn the picture referents of native-language pseudowords from word-picture mappings when multiple pseudowords co-occur with multiple pictures through cross-situational statistical relations both when they merely observe the words and pictures (Yu and Smith, 2007; Vlach and Sandhofer, 2014; Kachergis and Yu, 2018) and when they also attempt to select the correct picture each time they hear a word during training (Kachergis and Yu, 2018). Adults are able to learn word-picture mappings even in situations that contain a high amount of uncertainty, such as when one word is presented together with nine pictures in each trial, but when uncertainty increases, their learning becomes slower and less reliable (Smith et al., 2011). Adults can also learn cross-situationally minimal pairs that differ by one vowel or one consonant, although the learning of vowel minimal pairs is weaker than that of non-minimal pairs (Escudero et al., 2016).

An additional factor that affects learning words is speech production. Many previous studies have demonstrated the effectiveness of producing aloud the words to be learned. Both children and adults have demonstrated the ability to learn to recognize foreign words with five repetitions in a task that required them to produce the words they heard without knowing their meanings (Service et al., 2014). Words are more effectively learned when they are produced aloud in comparison to reading words silently (e.g., Hopkins and Edwards, 1972). This production has been suggested to benefit recognition memory by making the words produced aloud more distinctive than silently read ones (Dodson and Schacter, 2001; MacLeod et al., 2010). The distinctiveness of the produced words does not depend on input modality, as this effect has been discovered to occur when words were learned by reading their written forms and the test items were written text, as well as when words were learned by hearing and producing them and test items were heard spoken words (Forrin and MacLeod, 2016). Almost all studies with significant production effects have used within-subjects designs, whereas the production effect has not been significant in between-subjects designs. However, a meta-analysis by Fawcett (2013) noticed a moderate production effect between-subjects, indicating that the between-subjects production effects are consistent even though they might be too small to reach significance in individual studies.

One influential component of the production effect appears to be that it is self-referential by nature. Forrin and MacLeod (2018) demonstrated that recognition memory results were better for words that participants learned while hearing them spoken in their own voice from a previous recording than for words that were learned while hearing them spoken by someone else. The benefits of producing stimuli oneself are not only limited to saying words aloud, but also occur when musical melodies are produced on a piano, which indicates that auditory-motor interactions are beneficial for memory (Mathias et al., 2015). On the other hand, Mama and Icht (2016) suggest that the number of different encoding processes and the requirement for active production explain the effectiveness of the production. In their experiments, they analyzed words that were learned by listening to them and compared the effects of reading, speaking, and writing the words in addition to listening. Listening and writing includes the greatest number of different encoding processes and it was the most effective method of learning, which suggests that including different modalities and active production enhances learning most effectively. This is in line with the findings of Forrin and MacLeod (2018) that it is more efficient to learn words by hearing them spoken by someone while seeing them written than by merely reading the written words silently. Another factor that has been suggested to enhance memory for produced words is the added difficulty by delaying the production while learning (Mama and Icht, 2018).

Few studies have been published on the production effect in children and the findings are contradictory. Icht and Mama (2015) discovered that children in preschool benefited from producing words aloud when they memorized familiar and unfamiliar native-language words. In contrast, Zamuner et al. (2018) demonstrated that children recognized the meanings of pseudowords with native phonemes better when they had only heard them during training compared to when they only produced them. The inconsistent results suggest that different types of stimuli and tasks could modify the production effect. Icht and Mama (2015) used pictures and names of familiar and unfamiliar real objects in their study, whereas the stimuli used by Zamuner et al. (2018) comprised pictures of nonce animals and pseudowords that referred to them. Thus, the study design adopted by Zamuner et al. (2018) could have introduced a heavier cognitive load to memorize the visual stimuli, which could have increased the complexity of the learning task. In this case, their listening training task required the participants to memorize an association between a picture they could not have seen before and a novel pseudoword they heard twice, whereas their listening and producing task required them to memorize the same association while having to produce the pseudoword they heard only once.

Most of the production effect studies on adults have focused on recognition memory for native-language words. However, a study by Zamuner et al. (2016) used pseudowords with native phonemes. They demonstrated that adult participants learned to associate a pseudoword with a picture of a nonce animal better when they had produced the pseudowords vocally rather than only hearing them. There was also a difference in detecting the closest sounding target of a mispronounced pseudoword based on how the pseudowords were learned. If the pseudowords were learned only by hearing them, participants did not display mappings of the mispronounced pseudowords onto the most similar target. In contrast, if pseudowords were also produced during their learning, the participants eventually associated the mispronounced pseudowords with the most similar target, suggesting they generalized the learned representation to the most similar variations. In contrast to the results reported by Zamuner et al. (2016); Baese-Berk and Samuel (2016) have proposed that producing words aloud during foreign-language training hinders the learning of speech sounds. The contradicting results might stem from the different types of stimuli that were used in the experiments. Baese-Berk and Samuel (2016) used non-native syllables as their stimuli, whereas Zamuner et al. (2016) used pseudowords with native phonemes, suggesting that the familiarity of the phonemes affected the efficiency of producing the words. This is in line with the results by Kaushanskaya and Yoo (2011) that rehearsing words with native phonemes by producing them aloud was more effective in learning their meanings than rehearsing them silently, but in contrast, rehearsing words with non-native phonemes by producing them aloud was less effective than rehearsing them silently.

To summarize, previous studies have explored different aspects of word learning in children, but they have not directly compared the effectiveness of many different learning methods by using the same test set. Furthermore, there are very few studies on the production effect in children and their results are inconsistent. Some studies have demonstrated that children benefit from saying aloud the words they are learning (Icht and Mama, 2015) while other studies have established that only hearing the words without speech production constitutes a more effective way to learn (Zamuner et al., 2018). Due to these conflicting results, the present study investigates the effects of four different tasks on children’s learning of foreign words and aims to clarify the contradictory claims on speech production. We directly compare the effects of incidental learning, intentional learning with production, intentional learning without production, and cross-situational statistical learning. In addition, as learning foreign words requires learning the phonological representations and meanings of the words (Gupta and Tisdale, 2009), we examine the effects of the tasks on both the learning of word meanings and correct word forms.

This study has three main objectives. Firstly, our aim is to determine which of the four task types (incidental, intentional with production, intentional without production, cross-situational statistical) is the most effective for learning word meanings. We hypothesized that if children’s learning of foreign word meanings is similar to their learning the meanings of unfamiliar native words for real-life objects, that is, benefiting from producing them aloud (Icht and Mama, 2015), they would learn meanings of the words most effectively with the intentional learning task with production. Secondly, we aim to determine which of the four task types is the most effective for learning correct word forms. If children resemble adults in their learning of foreign word forms, we expected that vocal production would either hinder word-form learning, which is in line with Baese-Berk and Samuel (2016). or that production would enhance it, as reported by Zamuner et al. (2016). Thirdly, our aim is to analyze how the learning of meanings and word forms in different tasks is connected to children’s age or cognitive skills in order to determine whether these factors modulate the effectiveness of our tasks in the language-learning process. Our hypothesis was that children with better phonological or processing skills will learn word forms more efficiently (i.e., the phonological representations of words). We also expected to find that better phonological processing skills linked to a better ability to learn meanings. This assumption is based on previous findings that suggest that phonological sensitivity enhances the learning of unfamiliar words (de Jong et al., 2000) and that a better developed phonological awareness leads to more successful mapping of foreign words with their meanings in a cross-situational statistical learning setting (Hu, 2017). In particular, we assumed that a more developed phonological awareness improves the learning of meanings in the incidental task, given that previous studies have established a connection between them (Ramachandra et al., 2011; Abel and Schuele, 2014). Similarly, we expected that children with better working memory capacities, as indicated by the Digit Span task, would learn meanings more effectively in the intentional task, as the explicit learning of meanings has been reported to be connected to phonological short-term memory and working memory (Archibald and Joanisse, 2013). We incorporated the paired-associate learning task in our learning experiment and since the NEPSY-II (Korkman et al., 2008) Memory for Names test requires paired-associate learning as well, we hypothesized that children who perform better in the Memory for Names test would also learn the meanings more effectively. We also anticipated older children to be better learners. We specifically expected older children to learn word meanings better than young children in the cross-situational statistical task, which is in line with Venker (2019).

## Materials and Methods

### Ethics

The study was approved by the University of Helsinki Ethical Review Board in the Humanities and Social and Behavioral Sciences. Participation was voluntary. The participants’ guardian(s) signed a written informed consent and the participants themselves gave their oral informed consent before participating. The entire study was conducted in accordance with the Declaration of Helsinki. As compensation for participating, each participant was given a movie ticket.

### Participants

Forty-four voluntary 5–8-year-old children participated in the study. The youngest participants were in kindergarten and the oldest were in the second grade of a Finnish comprehensive school. To be included in the study, the children had to fulfill the following criteria: age of 5–8 years, Finnish-speaking monolingual, has not studied English, no language disorders, no learning disorders, no developmental disorders, no sensory processing disorders, no neurological diagnoses or head injuries. They also had to have normal hearing and normal vision, or vision that has been corrected to normal with eyeglasses. Four participants were excluded from the study. Two of them were excluded due to technical problems, one participant had a sensory processing disorder, and one participant did not want to complete the learning tasks. The remaining 40 participants (a mean age of 6 years 6 months, SD 12 months) were all monolingual Finnish-speakers and 21 of them were boys. According to their guardian(s), none of the remaining participants had language disorders, learning difficulties, or developmental disorders. All participants also had normal hearing and normal vision, with the exception of three participants who had their vision corrected to normal with eyeglasses. None of the participants had received formal education in any foreign language. To determine the level of language knowledge that they had acquired outside of school, the guardians were asked whether the children knew any foreign languages. They reported that none of the participants spoke foreign languages or understood simple spoken sentences in any foreign language. A total of 23 participants only knew a few words in one or more foreign languages: 21 knew less than ten English words (e.g., yes, no, thanks, dog, car), and 10 of them knew at most ten words in some other foreign languages (Swedish, German, Spanish, French, or Italian).

As the experiment with all four learning tasks was considered excessively long and demanding for children, they were randomly divided into two groups. Half of the children (*N* = 20, mean age 6 years 5 months, SD 11 months, range: 65–104 months, 13 boys) participated in two tasks (an intentional task with production and an incidental task) and the other half (*N* = 20, mean age of 6 years 6 months, SD 13 months, range: 65–103 months, 8 boys) participated in the remaining two tasks (an intentional task without production and a cross-situational statistical task). The two groups did not differ from each other in their cognitive skills (Table 1).

**TABLE 1:** A comparison of the age and cognitive skill scores (mean ± SD) of participants for the intentional task with production and the incidental task (Group 1) and participants for the intentional task without production and statistical task (Group 2).

	**Group 1**	**Group 2**	***t*-test**
Age (days)	2 350.20 ± 347.93	2 384.95 ± 397.69	*t* = −0.29, *p* = 0.77
Block design	10.40 ± 3.15	9.55 ± 3.07	*t* = 0.86, *p* = 0.39
Digit span	9.65 ± 2.50	10.35 ± 2.76	*t* = −0.84, *p* = 0.41
Phonological processing	9.85 ± 2.76	10.40 ± 2.30	*t* = −0.68, *p* = 0.50
Memory for names	9.80 ± 1.85	10.80 ± 3.00	*t* = −1.27, *p* = 0.21

### Stimuli

The stimulus material consisted of 10 pictures of animals, 10 auditorily presented English words for the animals (an elk, a fly, a hedgehog, a ladybird, a lynx, a whale, a snake, a squirrel, a wolf, and a worm) and their 10 pseudoword counterparts with one or several altered vowels, such as (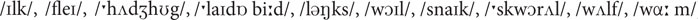). Although consonants are more important to lexical processing than vowels in most languages (Bonatti et al., 2005; Nazzi and Cutler, 2019), vowels are very frequent in Finnish [48% vowels, 52% consonants: See Vilkuna et al. (2004) (i.e., Descriptive Grammar of Finnish), §10], and therefore vowels have a more significant role in lexical processing in Finnish compared to many other languages (e.g., see Ylinen et al., 2017, for very distinct brain responses to Finnish spoken word forms with a vowel difference). For this reason, changed vowels were expected to be sufficiently distinctive for native speakers of Finnish in this task. Nevertheless, we cannot exclude the possibility that this task may have been more difficult than a task that presents consonant changes would have been, given the strong association between consonants and lexical processing (Nazzi and Cutler, 2019). The stimuli were recorded in a soundproof room where a native English speaker pronounced the words and pseudowords.

### Procedure

During the experiments, the children sat in a quiet room with the researcher. The experiments were conducted either at the participants’ homes, kindergartens, schools, or in the facilities of the University of Helsinki, whichever the guardian(s) preferred. Firstly, the children’s cognitive skills were assessed. After that, the training tasks were conducted and this was followed by the test phase. The duration of the experiment was 1 h.

Before beginning the learning tasks, we assessed the participants’ skills in memory tasks, as well as their phonological skills and perceptual reasoning skills in order to analyze the connections between them and foreign-word learning (to compare these skills between the two groups, see Table 1). The participants’ skills in memory tasks were assessed with the forward and backward Digit Span from the Wechsler Intelligence Scale for Children IV (WISC-IV; Wechsler, 2010) and for immediate recall and delayed recall, with the NEPSY-II (Korkman et al., 2008) Memory for Names. The Digit Span subtest measures auditory short-term memory. This consists of two parts: Digit Span Forward and Digit Span Backward. In both of them, the person conducting the task reads aloud a series of numbers and the child must then recite them from memory. In Digit Span Forward, the child must recite the numbers in the same order they were presented in. In Digit Span Backward, the child needs to recite the numbers in reverse order. The Memory for Names subtest assesses the participant’s ability to learn and remember the names of children and to associate these names and pictures of the children’s faces. The child is presented with cards that have drawings of other children’s faces. These are shown to the child one by one and the child is also told the children’s first names. The drawings are then immediately shuffled and presented again one by one, and this time, the child is asked to recall the names of the children. The names that the child cannot recall are told to them. This is repeated three times. The delayed recall is tested by repeating the task 25–35 min after the initial task. Phonological skills were assessed with the NEPSY-II Phonological Processing test. The Phonological Processing subtest assesses phonological awareness and includes three parts. The first part requires the child to identify words that are presented orally in segments or to identify words by only hearing one part of them. The second part requires the child to create new words by omitting phonemes or syllables from orally presented words. The third part requires the child to create new words by substituting segments of orally presented words with different segments. Perceptual reasoning skills were assessed with the WISC-IV Block Design test. The Block Design subtest assesses the ability to understand abstract visual information. The child is presented with a model that is either constructed from blocks or a model picture. Then they are required to construct a similar design using two colored blocks. The time required for the child to complete the test is then measured and a faster performance indicates better skills. This test was included in order to assess cognitive skills that are not related to verbal skills.

During training, the children practiced English words for animals on a tablet computer in four different behavioral learning tasks: an intentional task with production, an intentional task without production, an incidental task, and a cross-situational statistical learning task. One group of participants practiced English words for five animals during an intentional task with production and words for five different animals during an incidental task. The other group practiced words for five animals during an intentional task without production and words for five different animals during a cross-situational statistical task. The children were exposed to each word in one task only. To control for variation in difficulty of different words, the words used in each task were counterbalanced across participants. A half of the participants in the first group learned the words *elk*, *hedgehog*, *ladybird*, *snake*, and *wolf* during an intentional task with production and the words *fly*, *lynx*, *whale*, *squirrel*, and *worm* during an incidental task. The other half of the first group learned the words *fly*, *lynx*, *whale*, *squirrel*, and *worm* during an intentional task with production and the words *elk*, *hedgehog*, *ladybird*, *snake*, and *wolf* during an incidental task. The words were similarly balanced for the other participant group for the intentional task without production and the cross-situational statistical task. The two participant groups did not differ in age or cognitive skills (Table 1). In all tasks, the stimuli were spoken English words for animals and the corresponding pictures of those animals. The spoken words were presented through headphones at a comfortable sound pressure level and the pictures were presented on a tablet screen. During each task, the children were supposed to learn the English words for five different animals. All language-learning tasks were considered appropriate for the current age range as the types of tasks (including object naming and paired-associate learning) are used in standardized cognitive tests for this age range (WISC, NEPSY). The tasks may be considered to resemble natural learning situations that are encountered during infancy and early childhood (for cross-situational statistical learning, see Smith and Yu, 2008). Before beginning the learning tasks, the children were asked to name each animal in Finnish to ensure they knew them in their native language. All children were able to name each animal in Finnish. They were also asked if they could name them in English and none of them could name any of the animals in English before the leaning tasks.

**FIGURE 1:**
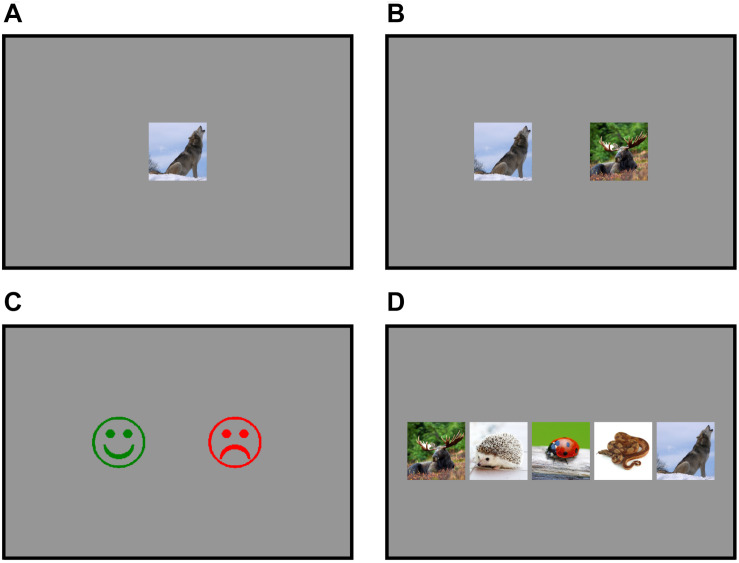
Examples of what the children were shown during learning tasks and tests. **(A)** During the intentional task with production, the intentional task without production, and the incidental task, the children heard an English word for an animal and a picture of the same animal was displayed on the screen. **(B)** During the cross-situational statistical task, the children were presented with pictures of two different animals: one that corresponded to the word they heard and another one that did not. **(C)** During the word-form test, the children responded by pressing an image of a smiling face and a sad face. **(D)** During the word meaning test, the children were presented with pictures of five different animals from the learning tasks, one of which corresponded to the word they heard.

In all tasks, foreign words and pictures of animals were presented in pairs, which allowed the children to associate them with each other. For example, first the spoken English word/w℧lf/was presented to them through headphones, and then the corresponding picture (a picture of a wolf) was presented to them on a tablet screen after 300 ms (Figure 1A). Each task included the names of five animals to learn and each animal and its name was presented ten times. The order of presentation was selected at random with the exception that none of the animals was presented twice in a row. During the intentional task with production, children were instructed to imitate the foreign words they heard and were then asked to remember them. In the intentional task without production, the children were instructed to try to remember the presented foreign words. During the incidental task, they were instructed to name the animal they saw in Finnish, but they were not asked to learn the English words they heard. In the cross-situational statistical task, the spoken word was followed by pictures of two different animals, one of which corresponded to the spoken word (Figure 1B), and the correct word-picture pairings could be deduced from the probability of co-occurrence across trials. The children were instructed to guess the correct animal, press the picture of that animal, and learn the association. To familiarize the participants with the task types and to ensure they understood the instructions, the participants performed practice trials after hearing the task instruction before beginning the actual training task. The animals that were used in practice trials were not used in any of the actual training tasks. The order of the learning tasks was counterbalanced between participants as were the words used in each task to control for variation in difficulty of different words.

After the children completed the different word learning tasks, they were tested for their learning of word forms and word meanings. The learning of word forms was tested by presenting the spoken learned words and their pseudoword counterparts by altering vowels, so that a word such as/wƱlf/ became /wʌlf/. Each of the ten correct trained words (five words for each of the two tasks) and their 10 pseudoword counterparts were presented once in a random order, one at a time. Simultaneously, pictures of a smiling face and a sad face were shown on the tablet screen (Figure 1C). The children were instructed to press the smiling face when the word they heard was one of the words they had learned and press the sad face when the word was not among the learned words. During the word meaning test, the children were presented with the learned words one at a time while they were shown the pictures of five different animals in a fixed order (Figure 1D). One of the animals was always the correct one, whereas the remaining animals were the other items that were previously presented in the same learning task. The children were instructed to press the picture of the correct animal on the touch screen. Each of the ten words (five words for each of the two tasks) was presented five times in a random order with the limitation that none of the words were presented more than once consecutively. All learning tasks and tests were presented by using Presentation software (Neurobehavioral Systems, version 18.1)^2^.

### Analysis

The statistical significance of the word-form test and meaning test scores (percentage correct) were compared to the chance level with one-tailed *t*-tests. To compare the learning results of the four different tasks in learning word forms or meaning, the data were analyzed using a linear mixed model with SPSS software (version 24.0.0.1). Correlation analyses (Pearson’s R) were also performed with SPSS software to analyze the connections between the learning results and age, Phonological Processing, Block Design, Digit Span, and Memory for Names. The Benjamini–Hochberg procedure (Benjamini and Hochberg, 1995) was conducted by using R programming language (R Core Team, 2018) to control the false discovery rate (FDR) for multiple comparisons. The *p*-values were adjusted for an FDR of 0.05.

## Results

**FIGURE 2:**
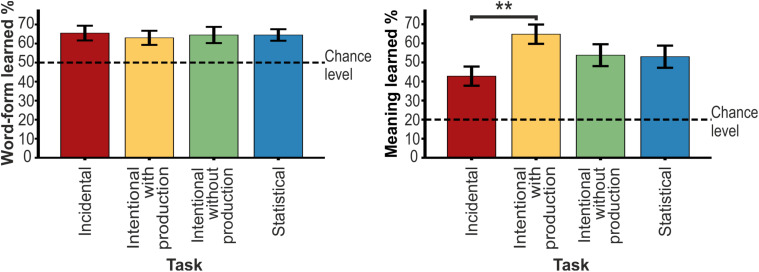
The mean scores and the *SEM* for learned word-forms and meanings for four different tasks. The significant difference is marked with asterisks. Chance level is determined by the number of response options.

The *t-*tests revealed that the children’s performance on the word-form test was above chance for all learning tasks (Table 2 and Figure 2). The children learned word forms during all tasks. According to the linear mixed model analysis, no task effect was found, *F*(3, 55.226) = 0.081, *p* = 0.970.

**TABLE 2:** *t*-tests comparing the percentage of word forms learned against a chance level of 50%.

	***M***	***SD***	***t***	***p***
Incidental	65.50	17.31	4.00	0.001***
Intentional with production	63.00	16.58	3.51	0.002**
Intentional without production	64.50	19.05	3.40	0.003**
Statistical	64.50	13.56	4.78	<0.001***

**p ≤ 0.01, ***p ≤ 0.001.

The children’s performance on the meaning test was above chance for all learning tasks (Table 3 and Figure 2). A significant effect of task was discovered with the linear mixed model analysis, *F*(3, 52.306) = 4.851, *p* = 0.005. Further pairwise comparisons revealed a difference between the incidental and the intentional task with production (*p* = 0.003). The percentage of learned words for the intentional task with production (64.8%) was higher than that of the incidental task (42.8%).

**TABLE 3:** *t*-tests comparing the percentage of word meanings learned against a chance level of 20%.

	***M***	***SD***	***T***	***p***
Incidental	42.80	22.48	4.54	<0.001***
Intentional with production	64.80	22.65	8.85	<0.001***
Intentional without production	53.80	25.64	5.89	<0.001***
Statistical	53.00	25.98	5.68	<0.001***

***p ≤ 0.001.

**FIGURE 3:**
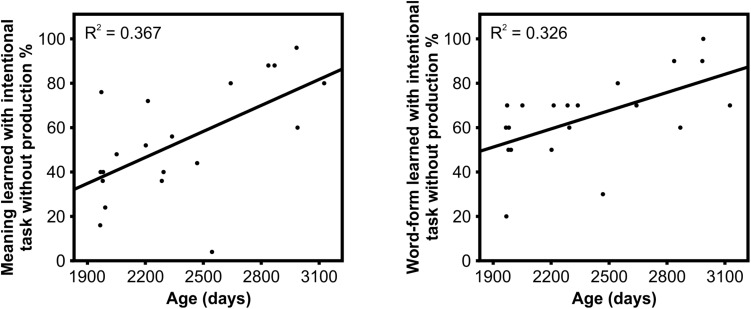
Correlations between age and learning the meanings (left) and correct word forms (right) for the intentional task without production.

For the intentional task without production, a correlation analysis indicated a significant positive correlation between age and learning the meanings [*r*(18) = 0.61, *p* = 0.034] as well as between age and word forms [*r*(18) = 0.57, *p* = 0.034] (Table 4 and Figure 3). Thus, the older the children were, the better they learned the meanings and word forms during the intentional task without production. No other correlations between age and learning outcomes were statistically significant.

**TABLE 4:** Correlations between age and learning performance with critical values of the Benjamini–Hochberg correction at an FDR of 0.05 and corrected *p*-values.

**Task (learning measure)**	***r***	**Raw *p*-value**	**Rank**	**Critical value at 0.05**	**Corrected *p*-value**
Intentional without production (meaning)	0.61	0.005	1	0.006	0.034
Intentional without production (word form)	0.57	0.009	2	0.013	0.034
Incidental (meaning)	0.50	0.024	3	0.019	0.063
Statistical (meaning)	0.42	0.065	4	0.025	0.131
Intentional with production (meaning)	0.35	0.137	5	0.031	0.219
Intentional with production (word form)	0.26	0.264	6	0.038	0.352
Statistical (word form)	0.21	0.371	7	0.044	0.423
Incidental (word form)	0.01	0.967	8	0.050	0.967

**FIGURE 4:**
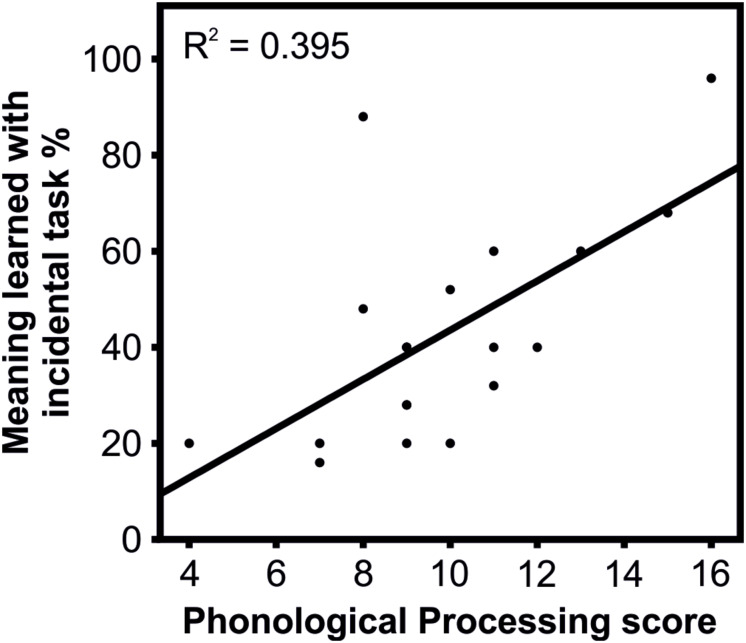
Correlation between the Phonological Processing score and learning the meanings for the incidental task.

The correlation analysis of cognitive skills and learning results revealed a significant positive correlation between Phonological Processing and learning the word meanings in the incidental task [*r*(18) = 0.63, *p* = 0.024]. Better phonological processing skills are thus connected to the better incidental learning of word meanings (Table 5 and Figure 4). No other correlations between cognitive skills and learning results were statistically significant (Tables 5–8).

**TABLE 5:** Correlations between Phonological Processing and learning performance with critical values of the Benjamini–Hochberg correction at an FDR of 0.05 and corrected *p*-values.

**Task (learning measure)**	***r***	**Raw *p*-value**	**Rank**	**Critical value at 0.05**	**Corrected *p*-value**
Incidental (meaning)	0.63	0.003	1	0.006	0.024
Statistical (word form)	0.28	0.238	2	0.013	0.637
Statistical (meaning)	0.24	0.303	3	0.019	0.637
Intentional without production (word form)	−0.24	0.318	4	0.025	0.637
Intentional with production (word form)	0.11	0.632	5	0.031	0.851
Intentional without production (meaning)	0.11	0.639	6	0.038	0.851
Intentional with production (meaning)	0.03	0.892	7	0.044	0.903
Incidental (word form)	0.03	0.903	8	0.050	0.903

**TABLE 6:** Correlations between Block Design and learning performance with critical values of the Benjamini–Hochberg correction at an FDR of 0.05 and corrected *p*-values.

**Task (learning measure)**	***r***	**Raw *p*-value**	**Rank**	**Critical value at 0.05**	**Corrected *p*-value**
Intentional with production (word form)	0.37	0.110	1	0.006	0.486
Incidental (meaning)	0.36	0.122	2	0.013	0.486
Statistical (meaning)	0.23	0.334	3	0.019	0.859
Intentional without production (word form)	−0.14	0.572	4	0.025	0.859
Incidental (word form)	0.13	0.582	5	0.031	0.859
Intentional without production (meaning)	0.10	0.669	6	0.038	0.859
Intentional with production (meaning)	−0.08	0.752	7	0.044	0.859
Statistical (word form)	0.00	0.998	8	0.050	0.998

**TABLE 7:** Correlations between Digit Span and learning performance with critical values of the Benjamini–Hochberg correction at an FDR of 0.05 and corrected *p*-values.

**Task (learning measure)**	***r***	**Raw *p*-value**	**Rank**	**Critical value at 0.05**	**Corrected *p*-value**
Incidental (meaning)	0.41	0.071	1	0.006	0.416
Statistical (meaning)	0.30	0.194	2	0.013	0.416
Statistical (word form)	0.29	0.209	3	0.019	0.416
Intentional with production (meaning)	0.28	0.237	4	0.025	0.416
Intentional without production (meaning)	0.26	0.260	5	0.031	0.416
Intentional without production (word form)	−0.14	0.551	6	0.038	0.662
Incidental (word form)	0.13	0.579	7	0.044	0.662
Intentional with production (word form)	0.01	0.953	8	0.050	0.953

**TABLE 8:** Correlations between the Memory for Names and learning performance with critical values of the Benjamini–Hochberg correction at an FDR of 0.05 and corrected *p*-values.

**Task (learning measure)**	***r***	**Raw *p*-value**	**Rank**	**Critical value at 0.05**	**Corrected *p*-value**
Incidental (word form)	−0.37	0.104	1	0.006	0.833
Intentional with production (meaning)	−0.13	0.581	2	0.013	0.884
Statistical (meaning)	0.10	0.684	3	0.019	0.884
Incidental (meaning)	−0.10	0.684	4	0.025	0.884
Intentional with production (word form)	0.09	0.709	5	0.031	0.884
Statistical (word form)	−0.05	0.820	6	0.038	0.884
Intentional without production (meaning)	0.04	0.875	7	0.044	0.884
Intentional without production (word form)	0.04	0.884	8	0.050	0.884

## Discussion

The purpose of this study was to determine which tasks would be the most effective for children in their learning of foreign word forms and word meanings, as these are needed to learn foreign languages. More specifically, we compared the effectiveness of incidental learning, intentional learning with production, intentional learning without production, and cross-situational statistical learning in children’s foreign-word learning. Furthermore, we examined the connections that meaning and word-form learning have to age and cognitive skills, including phonological processing skills, perceptual reasoning skills, and working memory capacity.

The learning performance for word meanings was above chance for each of the four different tasks. This means that the children were able to learn the associations between foreign words and the pictures depicting their meanings during all learning tasks used in the current study. There was a significant difference in learning scores between the incidental task and intentional task with production. Children were able to learn a higher number of words in the intentional task with production than in the incidental task. This suggests that the children’s foreign-word learning benefited from knowing that they were supposed to learn new words and produce them aloud while training. With respect to production effect, the results are consistent with the findings reported by Icht and Mama (2015) on children’s ability to learn familiar and unfamiliar native-language words.

Our pattern of results reveals that the intentional task with production has an advantage in the learning of word meanings compared to the incidental task, but no significant difference was detected between the intentional tasks with or without production. A possible explanation for not finding a difference between them is that in our study, different participants were involved in their learning of English words intentionally with production and intentionally without production. This means that this is a comparison between-subjects, where the effects have often been found to be too small to be significant in individual studies (Fawcett, 2013).

Similarly, to the learning of word meanings, the learning performance of correct word forms was also above chance for each of the four tasks. This indicates that children were able to learn to distinguish foreign word forms from minimal-pair pseudowords in each of the learning tasks. Unlike in the learning of word meanings, no differences were detected in the word-form learning performance between the tasks. In another words, we found no evidence that would suggest that any of these learning tasks benefits or hinders children’s word-form learning as compared to the other ones. This was inconsistent with previous studies on adults (Baese-Berk and Samuel, 2016; Zamuner et al., 2016), which may suggest that children and adults differ in their learning of word-forms. The differences in the results between the present study and the previous studies on adults (Baese-Berk and Samuel, 2016; Zamuner et al., 2016) may also originate from the different types of stimuli and test tasks used in the studies. The stimuli Baese-Berk and Samuel (2016) used were syllables from a continuum that was created from two foreign base-token syllables. Therefore, the phonemes in the stimulus pairs they used were very similar to each other, and thus pronouncing them might have interfered with learning to perceive their differences. In contrast, we taught children whole words with completely different phonemes. On the other hand, during their test phase, Zamuner et al. (2016) used pseudowords that differed from the learned word forms by their initial consonant. This introduces a shared rhyme between the learned word form and the mispronounced one used at the test. They tested learning effects with a task in which participants were shown two images side-by-side. The participants had to look at the one image that corresponded to the auditorily presented word. In this case, the mispronounced test word did not occur with a correct picture referent, but production during the learning phase could have caused a mispronounced word to activate the representation of the actual learned word form more strongly based on the shared rhyme.

Contrary to our hypothesis, no connection was found between phonological processing skills and the learning of word forms. However, phonological processing was connected to the learning of word meanings in the incidental task, as we hypothesized based on previous studies (Ramachandra et al., 2011; Abel and Schuele, 2014). This result suggests a link between phonological skills and learning the word meanings and it is also consistent with the findings of de Jong et al. (2000), who demonstrated that phonological sensitivity enhances the learning of unfamiliar words. The only task in the current study that was connected to phonological processing was the incidental task. This could indicate that individual differences play a larger role in an incidental learning setting. If this is the case, to benefit as many children as possible, regardless of their phonological processing skills, tasks other than incidental learning might be more successful as foreign-language training applications for children.

The children’s ages correlated with learning results for word forms and meanings in the intentional task without production. In that task, older children were more efficient in their learning of correct word forms and word meanings than younger children. Although older children outperformed the younger ones in the intentional task without production, the other three tasks that were more active were equally effective for all children regardless of age. Using active tasks can be beneficial for children’s ability to learn across the entire age range, not only the older children. Therefore, when designing foreign-language training programmes for children, it may be beneficial to design active learning tasks instead of tasks that do not require the learner to actively do anything but learn.

Children of different ages seem to rely on somewhat different skills and processes when learning foreign words. In contrast to our expectations that were based on previous studies, we did not find correlations between phonological processing skills and learning the meanings of words in the cross-situational statistical learning task (cf. Hu, 2017), or working memory capacity as indicated by Digit Span and the intentional learning task with or without production (cf. Archibald and Joanisse, 2013). It is important to note that we focused on a younger age group than Hu (2017) as well as Archibald and Joanisse (2013), and thus the discrepancy may indicate that different cognitive skills are connected to foreign-language learning at different stages of children’s development. Phonological processing skills in the cross-situational statistical learning and working memory capacity in intentional learning may not be strongly connected to foreign-word learning in 5–8 year-old children, but their role might increase with age.

The nature of stimuli appears to influence foreign-word learning considerably. Our results on the effectiveness of intentional learning with production as compared to incidental learning contradict those of Zamuner et al. (2018) who demonstrated that it was advantageous to learn words by only hearing them rather than producing them aloud. This difference might be a result of the different level of complexity between the studies. The stimuli in the present study were foreign words for familiar animals, whereas Zamuner et al. (2018) used novel words for nonce animals. Thus, in their study, both the words and the pictures were new to children, which increased complexity of the task and therefore increased the cognitive load. Similarly, the increased complexity introduced by pictures of novel objects could explain why, unlike a previous study by Venker (2019), we did not find a correlation between age and cross-situational statistical learning. Older children might perform better when the task is cognitively more demanding, but age may be less relevant when the stimuli are less complex.

We did not detect a correlation between Memory for Names and learning the meanings of the words, which could indicate that learning names is a special case of paired associate learning. This position is supported by the contrast between our results and those of de Jong et al. (2000). Although in both cases, the words to be learned were novel to the children, de Jong et al. (2000) taught these children novel names of cuddly toys, whereas we taught them English words for animals. That is, in our study the children learned words that have meanings and not names that refer to an individual (de Jong et al., 2000).

Other factors that might have influenced the different results between the studies relate to differences in the tasks and study designs. This could explain the inconsistencies between our findings and the previous studies regarding the superiority of the intentional task with production over the incidental task in the learning of word meanings (cf. Alipour Madarsara et al., 2015) and not finding a connection between phonological processing skills and learning meanings during the cross-situational statistical task (cf. Hu, 2017). For Alipour Madarsara et al. (2015), the difference between the learning groups was that the intentional group knew their learning was being tested and the incidental group did not, whereas in the present study, the incidental group practiced the words with a different task, that is, naming the animals in their native language. The main difference between the study by Hu (2017) and the present analysis is the timing of the tests. The participants in the study by Hu (2017) were tested immediately after each learning block and the last test occurred when 15 min had passed after the last learning block, whereas in the present study, the learning was tested only after learning all of the words involved in the different tasks.

## Conclusion

The results of the present study suggest that although children learn foreign word forms and meanings with incidental learning, intentional learning with production, intentional learning without production, and cross-situational statistical learning, the different learning methods are not equally beneficial for all individuals. The children learned the meanings of the words more effectively during the intentional task with production than in the incidental task. Phonological processing skills correlate with learning the meanings in the incidental task and age correlates with learning both word forms and meanings in the intentional task without production. As a final remark, for children in the age range of 5–8 years, the learning tasks that require active participation and speech production might support their foreign-language learning most efficiently, regardless of their cognitive skills.

## Data Availability Statement

The datasets generated for this study are available on request to the corresponding author.

## Ethics Statement

The studies involving human participants were reviewed and approved by University of Helsinki Ethical Review Board in the Humanities and Social and Behavioral Sciences. Written informed consent to participate in this study was provided by the participants’ legal guardian/next of kin.

## Author Contributions

KJ and SY designed the study. KJ collected and analyzed the data and wrote the manuscript. SY critically revised and edited the manuscript.

## Conflict of Interest

The authors declare that the research was conducted in the absence of any commercial or financial relationships that could be construed as a potential conflict of interest.

## Footnotes

1 Learning can be categorized as incidental or intentional (Laufer and Hulstijn, 2001; Hulstijn, 2003; Hulstijn, 2005; Service et al., 2014). Incidental learning can be described as learning something unintentionally, without attention directed toward the material to be learned and without knowing that the learning results for a certain type of information will be tested. In contrast, during intentional learning, learners are aware of what information they should learn. The use of the terms incidental learning and intentional learning overlap with that of implicit and explicit learning and these terms have sometimes been used interchangeably in the literature. Implicit and explicit learning are often linked to acquiring implicit and explicit knowledge, respectively (Hulstijn, 2005, 2012; Rebuschat, 2013). This study focuses on the nature of the learning task, not on the nature of the knowledge gained, and we therefore adopt the terms *incidental learning* and *intentional learning*.

2 www.neurobs.com
